# Dissolution of Lipid-Based Matrices in Simulated Gastrointestinal Solutions to Evaluate Their Potential for the Encapsulation of Bioactive Ingredients for Foods

**DOI:** 10.1155/2014/749630

**Published:** 2014-06-12

**Authors:** Yves Raymond, Claude P. Champagne

**Affiliations:** Food Research and Development Centre, Agriculture and Agri-food Canada, 3600 Casavant O., Saint-Hyacinthe, QC, Canada J2S 8E3

## Abstract

The goal of the study was to compare the dissolution of chocolate to other lipid-based matrices suitable for the microencapsulation of bioactive ingredients in simulated gastrointestinal solutions. Particles having approximately 750 *μ*m or 2.5 mm were prepared from the following lipid-based matrices: cocoa butter, fractionated palm kernel oil (FPKO), chocolate, beeswax, carnauba wax, and paraffin. They were added to solutions designed to simulate gastric secretions (GS) or duodenum secretions (DS) at 37°C. Paraffin, carnauba wax, and bees wax did not dissolve in either the GS or DS media. Cocoa butter, FPKO, and chocolate dissolved in the DS medium. Cocoa butter, and to a lesser extent chocolate, also dissolved in the GS medium. With chocolate, dissolution was twice as fast as that with small particles (750 *μ*m) as compared to the larger (2.5 mm) ones. With 750 *μ*m particle sizes, 90% dissolution of chocolate beads was attained after only 60 minutes in the DS medium, while it took 120 minutes for 70% of FPKO beads to dissolve in the same conditions. The data are discussed from the perspective of controlled release in the gastrointestinal tract of encapsulated ingredients (minerals, oils, probiotic bacteria, enzymes, vitamins, and peptides) used in the development of functional foods.

## 1. Introduction

A variety of ingredients are used to supplement foods in order to develop functional foods in the hope of reducing the risk of diseases [1]. Popular ingredients are minerals, fibres, probiotics, antioxidants, plant sterols, and omega-3 oils. There is also interest in prebiotics, peptides, and enzymes. In some instances, the ingredient must reach the intestines in a bioactive form. In the case of probiotic bacteria or enzymes, this requires maintaining viability/activity during processing and storage and after consumption [2, 3]. With other compounds, the ingredient can generate off flavours or become oxidized. In these instances, it is advisable to encapsulate the bioactives [4] and only allow their release in the gastrointestinal tract (GIT).

Many encapsulation techniques for probiotics or other bioactives are available [5, 6]. Using lipids for encapsulation has the advantage of providing barriers to oxygen, acid, and moisture, as well as not dissolving in foods. When the goal is to deliver a bioactive ingredient which is in a powder form to the GIT, two lipid-based encapsulation techniques are found: spray-coating and spray-chilling (also termed spray-cooling) techniques. In the first, the molten lipid is sprayed at the surface of the powder [7], while in the second, the powder is blended to the molten fat and the mixture is subsequently allowed to solidify, typically in a column of cold air.

With probiotics or some enzymes, dissolution of the lipid coating is undesirable in the stomach, because of the stressful effect of the acid environment [8]. In these instances, the protective coat must not melt at temperatures below 37°C, which leaves a small number of food-grade lipids. Palm kernel oil has been suggested as a matrix for spray chilling [9] and chocolate is now recognized as a good carrier for probiotics [10]. The pharmaceutical industry also uses waxes for the controlled release of bioactives [11], but there are no comparative studies of their dissolution in the GIT environments in comparison to chocolate or oils.

The goal of this study was to evaluate the dissolution of five lipid-based compounds in simulated gastric and duodenum solutions, in order to ascertain their appropriateness as microencapsulation matrices for dried bioactives.

## 2. Methodology 

### 2.1. Lipid Compounds

Six matrices were tested for their dissolution properties in simulated gastrointestinal solutions. Chocolate was the Côte d'Or 86% Cacao brand (Mondelez Intl, Halle, Belgium); the Côte d'Or label stated the following ingredient content, per 50 g of product: 27 g fat, 17 g carbohydrates (including 7 g fibres and 5 g sugar), and 5 g protein. Cocoa butter flakes were of Mycryo (Barry Callebaut) and the product was completely liquid at 37°C. It must be mentioned that chocolate, cocoa butter, and FKPO contain numerous fatty acids and that this prevents the product from having a specific melting point. The matrices subsequently have variable “solid fat content” as a function of temperature (Table 1). As data from Table 1 shows, the completely liquefied state at 37°C of the cocoa butter used in this study was typical of this product. Fractionated palm kernel oil (FPKO) was graciously provided by Barry Callebaut (Kraft Foods, Saint-Hyacinthe, QC, Canada). Unfortunately, the specific “% fat in solid state” to “temperature” properties of the FPKO product are proprietary. However, it can be stated that the completely liquefied state was reached between 42 and 44°C and that the range of temperatures that generates the solid states is much narrower than that with the cocoa butter. Beeswax USP grade and Carnauba wax were from Spectrum Chemical Manufacturing Co, (Gardena, CA, USA) and had an MP between 62–65°C and 82–85.5°C, respectively. Granular paraffin wax was from Acros Organic (New Jersey, NJ, USA) with an MP in the 50–57°C range.

### 2.2. Gastrointestinal Solutions

Compounds used to make the gastrointestinal solutions were K_2_HPO_4_ anhydrous ACS Grade (Bioshop Canada Inc, Burlington, ON, Canada), NaHCO_3_ (Fluka Biochemika, Oakville, ON, Canada), and KCl (Assured BDH, Toronto, ON, Canada), while Sigma Aldrich (Oakville, ON, Canada) was the supplier of the following: NaCl, mucin from porcine stomach type III, lipase from porcine pancreas type II, pepsin from porcine gastric mucosa, alpha-amylase type VI-B from porcine pancreas, and pancreatin from porcine pancreas. Gastric secretion (GS) and duodenal secretion (DS) were prepared according to Table 2. Those composition parameters were adapted from those used in an in vitro digestive system (IViDiS) [12]. The pH values of GS and DS were of 1.5 and 6.8, respectively.

### 2.3. Production of Lipid Particles

Two series of fat or wax particles sizes were prepared which had, on the average, 2.5 mm or 0.75 mm diameters. For the preparation of the 2.5 mm beads, the lipid matrices were melted by adding solid particles into a test tube placed in a water bath (100°C). Particles were then poured into a syringe equipped with an 18 G 11/2 (BD) needle. The molten lipids were dripped on a stainless steel surface placed on crushed ice cubes on which they solidified. With respect to the preparation of particles having 750 *μ*m, the molten lipid in the syringe having the 18 G 11/2 needle tip was manually extruded and droplets fell into liquid nitrogen. The liquid nitrogen and fat particles were poured in a mortar and then crunched with the pestle until small granules were obtained. Once all the liquid nitrogen was evaporated, the particles were successively sieved with 1000 microns and 500 microns filters (Canadian Standard Sieve series, W.S. Tyler, St. Catharines, ON, Canada). The particles which had passed the 1000 *μ*m mesh filter but which were retained by the 500 *μ*m filter were kept and this product will be referred to as the “750 *μ*m particles.” In all cases (2.5 mm and 750 *μ*m sizes), the particles recovered were stored at 4°C until they were used.

### 2.4. Dissolution of Lipid-Base Matrices in Simulated Gastrointestinal Solutions

The various particles were placed into solutions that simulated gastric secretions (GS) and duodenal secretion (DS). Beads (0.8 g) were added to 7.2 mL of DS solutions or 3.08 mL of GS solutions (already at 37°C) in 15 mL conical tubes. The volume of the DS used (7.2 mL) was greater than that of the GS (3.08 mL) in order to represent the actual situation in the GIT. Indeed, as the food matrix containing the gastric ingredients passes from the stomach to the duodenum, the total volume of the digestate increases due to addition of the pancreatic juice and bile salts.

Five tubes were installed on a Cole-Parmer (Vernon Hills, IL, USA) Roto-Torque unit which carried out a gentle blending by inversion of the tubes at 55 rpm. The unit was placed in an incubator at 37°C. At given incubation times, a tube was taken and the quantity of dissolved particles, from the original 0.8 g, was estimated. Test tubes were first submitted to a vortex for 5 seconds. The suspensions were poured in a 250-micron stainless steel filter (Canadian Standard Sieve) preheated at 37°C and the filtrate was recovered.

### 2.5. Estimation of the Particle Dissolution Level

The level of dissolution was assessed by carrying out dry weights on the filtrate. Aluminium weighing dishes were first dried in the oven at 105°C for 30 minutes and put in a desiccator at room temperature for 20 minutes. The dry dishes were removed from the desiccator and weighed. The filtrate was then added in the dish and it was weighed again. The dishes were placed 22 h in the 105°C oven and then put in the desiccator at room temperature for 20 min and weighed. Controls of GS and DS solutions without beads were done in order to exactly ascertain the contribution of the dissolved lipid to the overweight. In some chocolate samples, the tubes were poured into the Buchner unit in which Whatman papers having 53 *μ*m or 8 *μ*m thresholds had been placed.

### 2.6. Statistical Analyses

Each dissolution treatment was repeated at least 3 times in an independent fashion (different days, fresh media, media sterilized independently, and so forth). Results are the average of these independently carried out triplicates. Statistical analyses were carried out using InStat (GraphPad, La Jolla, CA, USA) software.

## 3. Results

For all products, two sizes of beads were prepared in order to assess the effect of this parameter on dissolution. The 250 *μ*m mesh filters retained the lipid particles when the volume of the bead was reduced by less than 99% or 96%, for beads originally having 2.5 mm or 750 *μ*m, respectively (Table 3).

### 3.1. Cocoa Butter

The 2.5 mm cocoa butter particles dissolved in water alone within 30 minutes at 37°C (data not shown). Therefore, data were not presented in the figures on GS media and the product was not tested in the DS broth. Of the six lipid matrices tested, the cocoa butter particles were the least effective in preventing dissolution in simulated gastrointestinal conditions. Obviously, if one actually desires dissolution of the particles in the stomach, then the cocoa butter was the best of the matrices studied.

### 3.2. Dissolution in the Gastric Solution

**Figure 1:**
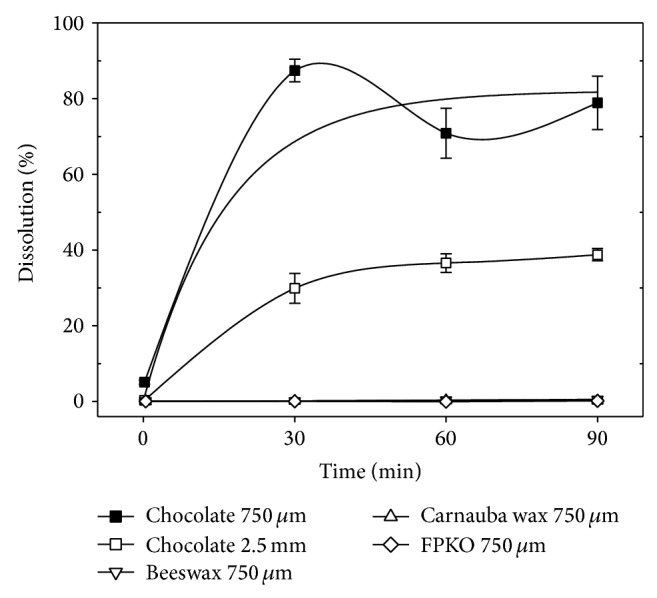
Effect of the matrix and particle size on dissolution in the simulated gastric solution (GS). Results are the average of three independent assays. Error bars represent Standard Error of the Means (SEM).

Small particles (750 *μ*m) were tested first for digestion in the GS and DS. In the GS, no dissolution was observed for beeswax, carnauba wax, and FPKO. For chocolate, a substantial dissolution was noted, which principally appeared in the first 30 minutes (Figure 1).

Tests were then made with the big 2.5 mm particles. It was assumed that small particles would dissolve more rapidly than the larger ones, and this was the case with chocolate, where liquefaction was 2.5 times lower with the 2.5 mm particles than that observed with the 750 *μ*m ones (Figure 1).

Since the small particles of paraffin, beeswax, carnauba wax, and FPKO did not dissolve in the GS, the large 2.5 mm beads of these matrices were not tested in this medium.

### 3.3. Dissolution in the Duodenum Solution

Many studies carry out dissolution assays in the duodenum by only testing the effects of pancreatic solutions and bile salts, which are the major compounds added at the duodenum level. Although this is useful in separately assessing the effects of these duodenum-specific ingredients, this approach does not represent the actual conditions in the GIT. In this study, it was therefore decided to include the simulated saliva and gastric compounds in the DS medium (Table 2).

**Figure 2:**
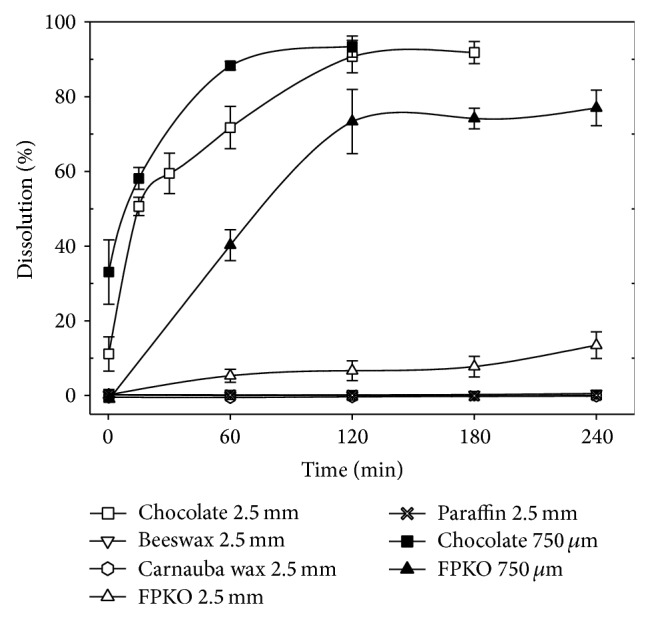
Effect of the matrix on dissolution of lipid-based particles in the simulated duodenum solution (DS). Results are the average of three independent assays. Error bars represent Standard Error of the Means (SEM).

In the DS (Figure 2), beeswax, paraffin, and carnauba still did not dissolve, irrespective of bead size. However, the dissolution level of 750 *μ*m chocolate particles appeared 11% higher than that in GS. Again, there was an effect of particle size in the chocolate dissolution rates, the larger beads being slower to liquefy (Figure 2).

FPKO was the product that had the most different response between the two media. Extensive dissolution was reached with the 750 *μ*m particles after 120 minutes in the DS (Figure 2), while none was noted in the GS (Figure 1). When the big beads of FPKO were placed in the DS, dissolution rate was reduced by a factor of almost 5. The lower dissolution level of the 2.5 mm FPKO particles, compared to 750 *μ*m ones, could be explained by a 1.7x lower surface to volume ratio exposed to the dissolving elements of DS (lipases, bile salts). Particle size also affected dissolution of chocolate beads in the DS, but the effect of particle size was not as important as that for FPKO.

### 3.4. Effect of Filter Size on Apparent Dissolution Levels of Chocolate

The chocolate product we used had 24% fiber and protein, a sizeable fraction potentially being insoluble. Indeed, grinding of cocoa beans results in many products, one of which is called the “nonfat powder” although it does contain a small fraction of fat. Particles sizes of this cocoa “nonfat component” vary from 0.2 to 100 *μ*m but are mostly in the 30 to 50 *μ*m range [13]. Of all the matrices used in this study, chocolate was the only one which had ingredients other than the lipids. It was therefore examined if results might be linked to the methodology used. In light of the potential presence of cocoa powder particles having 0.2 to 100 *μ*m sizes in the chocolate matrix, it was hypothesized that these cocoa particles could be retained in the smaller 8 *μ*m filters but not in the 250 *μ*m ones. Therefore, a series of chocolate dissolution analyses were carried out in DS in which a range of filters was tested (Table 3). With chocolate, the apparent dissolution values increased with filter pore size. Evidently this methodology affected data for chocolate, where nonfat cocoa particle retention by the filter occurred with the smallest pore size membranes. It can be argued that insoluble nonfat cocoa material had been released from the beads but was retained in some filters. Therefore, with chocolate, the 250 *μ*m filters were best to evaluate chocolate melting, and data at Figures 1 and 2 were obtained with the 250 *μ*m sieves.

## 4. Discussion

Encapsulation is carried out for many food ingredients in order to prevent their inactivation in the food matrix and to subsequently allow their delivery in the GIT [14]. Therefore, in some instances it can be undesirable to allow bioactive compounds to be in the soluble form in the functional food matrix during manufacture or storage of the product. In yoghurt, for example, it can be advantageous to encapsulate probiotics to limit their exposure to the acid environment [2]. Lipids are not soluble in aqueous environments. They, therefore, have potential to encapsulate bioactives for this purpose. By using spray-coating or spray-chilling technologies, it is possible to encapsulate dried hydrophilic bioactives (peptides, probiotics, and minerals) in fat matrices [15]. However, if the bioactives are added to foods for their health functions, the capsules must be able to release the hydrosoluble bioactives in the GIT.

There are various “triggers” to enable the dissolution of capsules [16]. In this study we will use melting point and GIT ingredients for this purpose. It is important to state that two GIT ingredients are not only useful for breakdown of the particle but also they allow solubilisation of the fat in the water phase. Indeed, bile salts are emulsifiers, and lipases generate free fatty acids from triglycerides which are water soluble at a pH of 7.0 when the free carboxylic end of the fatty acid chain is ionized (COO^−^). It is on this basis that the analytical method used in this study can assess lipid particle dissolution.

The food matrix has important effects on the bioavailability and functionality of health-promoting bioactives, for both hydrophilic [17, 18] and hydrophobic bioactives [19]. It is therefore useful to examine how some encapsulation matrices will potentially deliver bioactives having various patterns of solubility.

It was known that waxes did not dissolve well in gastrointestinal media [11] and these data confirm data in the literature. Waxes can still be used in pharmaceutical applications to slow the delivery systems; in these instances, since waxes do not dissolve to rapidly release the compounds and slow release occurring by simple diffusion [11, 20], presumably, in food systems, this could be useful for the slow release of omega-3 oils and lipophilic vitamins (A, D, E, or K) in the food itself or in the GIT.

Data from this study suggest that if powders of probiotic bacteria, long chain prebiotics (e.g., inulin), peptides, enzymes, or minerals (Ca, Se) were to be coated with paraffin, beeswax, or carnauba wax, they would presumably not be released in the stomach or the duodenum. Spray-coating technologies for the encapsulation of probiotics mostly employ lipids. Chocolate, cocoa butter, and FPKO particles broke down in the gastrointestinal solutions used in this study, albeit at various rates (Figure 2). Presumably, the low melting point of the cocoa butter matrix (Table 1) resulted in its disaggregation under the 37°C experimental conditions. However, the FPKO fat did not melt at 37°C. Therefore, complete breakdown in an aqueous environment would require the action of lipases and bile salts. Evidently the integrity of the FPKO particles was not affected in the GS medium, presumably because of the absence of bile salts and because the gastric lipase was not sufficiently active in hydrolysing the FPKO triglycerides.

These data suggest that chocolate, FPKO, and cocoa butter could be used as lipid-based matrices for application in either spray-coating or spray-chilling microencapsulation technologies of bioactive ingredients in the powder form. Since chocolate, cocoa butter, and FPKO had different dissolution rates in gastrointestinal solutions, the powder components inside the lipid matrix would also, presumably, be released at variable rates in the GIT. Selection of the lipid matrix would therefore allow for controlled release in the stomach or the intestine as a function of the most desirable site. For example, with respect to most applications of probiotic bacteria where a high delivery level of viable cells in the small intestine is desirable, small particles of FPKO (750 *μ*m) would be better than those of chocolate or cocoa butter. This is because no dissolution of FPKO occurs in the stomach, which is an environment highly detrimental to bacterial viability [2]. However, if bioactives (peptide, probiotic) would preferably be released in the stomach, for example, to fight* Helicobacter pylori* infections [21], then cocoa butter would be better than chocolate or FPKO. Waxes would be useless for any of these applications, because they do not dissolve the GIT environment, which would prevent the release of the bioactives in powder particles (probiotics, peptides, and minerals). However, waxes could be considered for very slow release of functional oils (omega-3 or omega-6 oils) in the duodenum. Indeed, the oils could simply diffuse outside the wax particle without requiring breakdown of the wax matrix as is done in some pharmaceutical applications [11].

Data of this study point to the importance of particle size on dissolution. With chocolate, dissolution was at least twice as fast as that in the GS and DS media with small particles (750 *μ*m) as compared to the larger (2.5 mm) ones. The impact of bead size in dissolution of FPKO in DS is even greater. As mentioned previously, the 750 *μ*m particles had a 1.7x higher ratio of surface to volume. Evidently, the greater the surface exposed to DS and GS solubilizing agents (bile and lipases) is, the faster the beads dissolved. These data point to the importance of characterizing particle size of microencapsulated bioactives when studying their release in the GIT. Unfortunately, this is often omitted.

Evaluating the dissolution of particles by dosing solutes in a filtrate is an easy methodology, but it has disadvantages. With chocolate, the method used to evaluate dissolution affected results. The methodology based on filtration to remove undissolved particles is best used with pure lipid compounds.

## 5. Concluding Remarks

Bile salts and lipases are compounds in the GIT which could allow the breakdown of fat matrices in an aqueous environment. This study's main contribution to the field is the comparative dissolution of waxes and chocolate-based products in gastrointestinal environments.

With particular reference to probiotic bacteria, since cocoa butter dissolves very rapidly and chocolate shows partial dissolution in the stomach environment, they would potentially release the cells too early. Indeed viability loses occur in the stomach due to high acidity and enzyme [22], and means to protect the cultures need to be developed. Therefore, of the chocolate, cocoa butter, and FPKO matrices, FPKO is the one which solely dissolved in the duodenum and which would presumably release the bioactives at that level. Therefore of the various matrices tested, FPKO appears to be the most suitable to be used for the encapsulation of probiotic bacteria. Studies in dynamic in vitro systems [8, 12] are currently underway to assess if these presumptions are correct and if microencapsulation enhances the survival of probiotics to passage through the stomach.

## Figures and Tables

**Table 1:** Solid fat content of cocoa butter (adapted from the data of Beckett [13]).

Temperature (°C)	Solid fat content range (%)
20	66–81
25	60–76
30	37–55
32.5	7–20
35	0–2

**Table 2:** Composition of the solutions used to prepare the simulated gastrointestinal solutions and amounts required to prepare the gastric secretion (GS) and duodenum secretion (DS) media.

	Saliva	Basal gastric secretions	Active gastric secretions	Basal pancreatic secretions	Lipase	Active pancreatic secretions	Bile
Composition	6 mM KCl	20 mM HCl	15 mM KCl	100 mM NaCl	0.9 mM HCl	20 mM NaCl	100 g/L Oxgall
	62 mM NaHCO_3_	10 mM KCl	5 mM NaCl	50 mM NaHCO_3_	10 mM KCl	140 mM NaHCO_3_	
	15 mM NaCl	90 mM NaCl	150 mM HCL	10 mM KCl	90 mM NaCl	10 mM KCl	
	6 mM K_2_HPO_4_·3H_2_O	100 U/mL Pepsin	1000 U/mL Pepsin	1.2 g/L pancreatin	300 U/mL lipases	12 g/L pancreatin	
	0.0066 mM CaCl_2_·2H_2_O						
	2.2 g/L Mucin						
	2000 U/mL *α*-amylase						

Amount of GS medium (mL)	39	16	35		11		

Amount of DS medium (mL)	17	7	15	3	5	37	17

**Table 3:** Effect of filter pore size on the apparent solubilisation of chocolate (in %) in DS medium.

Filter pore size	Bead size	
	750 *μ*m	2.5 mm
250 *μ*m	95%	92%
53 *μ*m	8%	56%
8 *μ*m	35%	27%
